# Trajectories of arm recovery early after stroke: an exploratory study using latent class growth analysis

**DOI:** 10.1080/07853890.2022.2159062

**Published:** 2023-01-03

**Authors:** Angela Vratsistas-Curto, Aron Downie, Annie McCluskey, Catherine Sherrington

**Affiliations:** a Institute of Musculoskeletal Health, School of Public Health, The University of Sydney, Sydney, Australia; b Health and Human Sciences, Faculty of Medicine, Macquarie University, Sydney, Australia; c Sydney School of Health Sciences, Faculty of Medicine and Health, The University of Sydney, Sydney, Australia; d StrokeEd Collaboration, Sydney, Australia

**Keywords:** Stroke, stroke rehabilitation, arm, prognosis, upper extremity, latent class growth analysis

## Abstract

**Aim:**

To investigate trajectories of recovery of motor arm function after stroke during inpatient rehabilitation.

**Materials and methods:**

Data were available from 74 consecutively-admitted stroke survivors receiving inpatient rehabilitation from an inception cohort study. Heterogeneity of arm recovery in the first 4-weeks was investigated using latent class analysis and weekly Box and Block Test (BBT) scores. Optimal number of clusters were determined; characterised and cluster associated factors explored.

**Results:**

A 4-cluster model was identified, including 19 participants with low baseline arm function and minimal recovery (‘LOWstart/LOWprogress’, 26%), 15 with moderate function and low recovery (‘MODstart/LOWprogress’, 20%), 15 with low function and high recovery (‘LOWstart/HIGHprogress’, 20%), and 25 with moderate function and recovery (‘MODstart/MODprogress’, 34%). Compared to LOWstart/LOWprogress: LOWstart/HIGHprogress presented earlier post-stroke (*β*, 95%CI) (−4.81 days, −8.94 to −0.69); MODstart/MODprogress had lower modified Rankin Scale scores (−0.74, −1.15 to −0.32); and MODstart/LOWprogress, LOWstart/HIGHprogress and MODstart/MODprogress had higher admission BBT (23.58, 18.82 to 28.34; 4.85, 0.85 to 9.61; 28.02, 23.82 to 32.21), Upper Limb-Motor Assessment Scale (9.60, 7.24 to 11.97; 3.34, 0.97 to 5.70; 10.86, 8.77 to 12.94), Action Research Arm Test (31.09, 22.86 to 39.33; 12.69, 4.46 to 20.93; 38.01, 30.76 to 45.27), and Manual Muscle Test scores (10.64, 7.07 to 14.21; 6.24, 2.67 to 9.81; 11.87, 8.72 to 15.01).

**Conclusions:**

We found unique patterns of arm recovery with distinct characteristics for each cluster. Better understanding of patterns of arm recovery can guide future models and intervention development.

**KEY MESSAGES**
- Arm recovery early after stroke follows four distinct trajectories that relate to time post stroke, initial stroke severity and baseline level of motor arm function.
- Identification of recovery patterns gives insight into the uniqueness of individual’s recovery.
- This study offers a novel approach on which to build and develop future models of arm recovery.

## Introduction

Motor arm impairment is common after stroke, affecting between 50% and 70% of people in the first week [1–3]. In Australia the median hospital length of stay is 5 days in acute wards and 22 days in rehabilitation wards [4], giving a combined hospital length of stay of approximately one month. Making predictions about arm function recovery during the first month after stroke is a challenge for clinicians, and creates uncertainty for stroke survivors. While there is a growing body of research, more still needs to be understood about arm recovery early after stroke.

Systematic reviews have identified a number of clinical and neurological biomarkers (i.e. neuroimaging and/or neurophysiological measures of brain) that are associated with reduced or increased arm recovery post-stroke [5–7]. Clinical factors include low levels of baseline arm impairment, higher baseline arm function, and lower levels of leg impairment [7]. Neurological biomarkers include various measures of corticospinal integrity using Transcranial Magnetic Stimulation, Diffusor Tensor Imaging or Magnetic Resonance Imaging [5,7]. A small number of arm recovery models have been proposed using clinical and neurological biomarkers. These models include SAFE (Shoulder Abduction-Finger Extension) [8], and Proportional Recovery [9], which both include clinical factors found to be predictive of arm recovery. The PREP and PREP2 models (predicting recovery potential) include both clinical factors and neurological biomarkers found to be associated with arm recovery [10,11].

There are limitations with these three arm recovery models. First, some studies used small (up to 41 participants) [9,11] or non-consecutive samples [8]. Second, a significant proportion of cases deemed to be outliers were excluded from analysis during the modelling process [9]. Third, the studies included specific stroke types, such as ischaemic [8,9] or anterior circulation strokes [11], excluding participants with other stroke types (for example haemorrhagic stroke) who also experience arm impairment. Fourth, participants with significant cognitive and/or language impairment were typically excluded; these participants represent an important sub-group of stroke survivors [8,11]. Fifth, the measurement of outcomes occurred at limited time points, such as 3- and 6-months post-stroke, providing a limited picture of arm recovery over time. Finally, many models use population averaged recovery which may not adequately reflect an individual’s course. These limitations raise questions about whether the models are representative of and applicable to, the broader stroke population [5–7].

Studies suggest that inclusion of neurological biomarkers improves the predictive ability of arm recovery models [10,11]. However, there is a lack of robust evidence that models with neurological biomarkers are statistically superior in their predictive ability to models with clinical factors alone [12]. Many neurological biomarkers involve expensive investigative procedures (e.g. MRI) and/or cannot be completed routinely with all hospitalised stroke survivors. Transcranial magnetic stimulation (TMS) is used in PREP and PREP2 to assess the presence of motor evoked potentials (MEPs), however many rehabilitation therapists are not trained in TMS and do not have the necessary equipment to complete the TMS assessment.

With the exception of PREP and PREP2, previous models have not accounted for heterogeneity in arm recovery post-stroke. Identification of unique recovery trajectories is likely to be an important factor for (i) developing better models of arm recovery and (ii) developing better interventions. Latent class growth analysis (LCGA) is one method to identify meaningful subgroups or classes of individuals that are homogenous amongst a heterogenous population [13] and has been applied in psychological [14] and musculoskeletal research [15]. Latent class growth analysis classifies participants into a finite number of clusters based on recovery trajectory. Participants within each cluster can then be described and compared to other clusters using baseline characteristics.

Latent class growth analysis is being increasingly used in stroke research to investigate trajectories of quality of life [16], functional recovery [17], care needs [18], psychological recovery [19], and post-stroke fatigue [20]. Only one known study to date has used LCGA to predict trajectories of arm recovery in the first 12 months after stroke using elbow kinetic and kinematic measures [21]. They found 2 classes of arm recovery where in the first cluster, participants had low baseline arm function (indicated by a low Fugl-Myer score) a rapid improvement before tapering off gradually [21]. In the second cluster, participants had high baseline arm function which remained constant or increased slightly [21]. The advantages of LCGA over other statistical methods include the ability to explain heterogeneity in longitudinal data, handle missing data points and outliers without their exclusion [22].

We used LCGA to explore trajectories of arm recovery early after stroke (in the first month) during inpatient rehabilitation. Trajectories were explored using an outcome measure which could be easily assessed at the bedside (i.e. Box and Block Test) measured at more frequent intervals (i.e. weekly).

The aims of this study were to identify trajectories of arm recovery in stroke survivors receiving inpatient rehabilitation in the first month after stroke, and explore characteristics associated with cluster membership. We hypothesised that cluster membership would be associated with clinical and demographic characteristics measured at baseline/early after admission.

## Materials and methods

Data were obtained from an inception cohort study briefly described below and in detail elsewhere [23].

### Study data

The original inception cohort study was conducted in comprehensive stroke unit in Sydney, Australia [23]. Acute and rehabilitation beds are co-located on the unit, with stroke survivors commencing rehabilitation early after admission [23]. In this setting, participants were able to be observed throughout their entire hospital stay. A consecutive cohort of eligible stroke survivors were approached on admission to the unit and written consent obtained to participate in the study [23]. The study was approved by the local health district ethics committee (LNR 11 L/144) [23]. Participants included in the study had a confirmed diagnosis of stroke, stroke related arm impairment, a score of <18 on the Upper Limb-Motor Assessment Scale (Items 6, 7 and 8) on admission and a score of ≥3 on the Modified Rankin Scale [23]. Those unable to participate in an arm rehabilitation program (i.e. medically unstable) or lacked sufficient cognition and communication (based on a score of less than 4/6 on the Six Item Screener [24]) were excluded from the study [23].

Baseline variables were collected early after admission *via* physical assessment or from the medical record. Arm measures completed on admission and discharge included the Upper Limb-Motor Assessment Scale (UL-MAS), Manual Muscle Test (MMT), Action Research (ARAT) Arm Test and Self-Care items on the Functional Independence Measure (FIM). The primary outcome measure was the Box and Block Test (BBT) which was completed weekly over each participant’s hospital stay. The BBT, UL-MAS and ARAT were used to detect change in motor arm function, MMT was used to detect changes in muscle strength and FIM-Self Care was used to detect change in functional activities involving arm use.

Repetitions of arm practice were counted and recorded prospectively by the stroke participant or treating therapist. Data about the amount and repetitions of arm practice completed during each participants hospital stay were collected prospectively from their medical record. A study had previously been conducted in the same setting showing that therapists can identify rehabilitation inpatients who were capable of accurately counting repetitions of practice completed [25]. All participants completed task-specific arm retraining as part of their usual rehabilitation program, consistent with Australian guideline recommendations [26]. Detailed information on the amount (repetitions and sessions completed), type (arm group, physiotherapy group, dressing group, one-to-one occupational therapy and independent practice) and examples of arm practice completed are reported elsewhere [23].

### Analysed participant data

Our primary outcome was motor arm recovery measured by the Box and Block Test, collected on admission and after 1, 2, 3 and 4 weeks unless the person had been discharged. Variables collected *via* physical assessment or from the medical record early after admission and analysed, included age (years), gender (male versus female), type of stroke (infarct versus haemorrhage), side of stroke (right versus left), co-morbidities (Charlson Comorbidity Index [27] of ≥1), stroke severity (modified Rankin Scale [28]), admission Upper Limb-Motor Assessment Scale score [29,30], admission Manual Muscle Test score [31–33], admission Box and Block Test score [34] and presence of spasticity (admission Tardieu Scale score of ≥2) [35,36]. Data on the dose (repetitions) of arm practice completed in the first week of admission collected as part of the inception cohort study were also used in the analysis. Some clinical variables were selected because they are known to be associated with arm recovery, for example, age, stroke severity and initial arm impairment/function [7,12]. We hypothesised spasticity to be associated with reduced arm recovery. Several systematic reviews and meta-analyses have shown that increasing the dose of rehabilitation can lead to better outcomes after stroke [37–39]. We therefore hypothesised that increased arm practice dose (repetitions) would to be associated with greater arm recovery and predict cluster membership.

### Data analysis

Statistical analyses were conducted to [1] identify unique trajectories of arm recovery and [2] characterise cluster membership using variables collected at baseline/soon after admission. Statistical analyses were completed using Mplus version 8 (Muthén & Muthén, California, United States), Excel 2010 (Microsoft, New Mexico, United States) and Stata v16.1 (StataCorp, Texas, United States). The main assumption in our analysis was that a number of distinct unobserved trajectory subgroups (clusters) for arm recovery exist, indicated by the patterns of Box and Block Test scores measured across five time points. Latent class growth analysis (LCGA) was used to identify unique clusters which explain heterogeneity in the data over time, then assign participants to a single cluster based on their probability of belonging. Regression analyses compared characteristics of participants assigned to each cluster against the cluster with the lowest progression. An alternative latent variable modelling approach (growth mixture modelling) was also considered, but demonstrated poorer model fit [13,40]. Guidelines for Reporting on Latent Trajectory Studies (GRoLTS) were followed for model selection [41] (Appendix 1).

Arm recovery was modelled over time using weekly Box and Block Test scores collected during the first month of admission on the stroke unit (baseline, weeks 1, 2, 3 and 4 weeks). Box and Block Test scores had a non-normal (heavy-tailed) distribution so were grouped into quartiles for each time point. This was done to decrease the potential for misclassification of participants in the latent model [42,43], and has been used previously [44–46]. We examined the pattern of missing Box and Block Test scores at each time-point, to find that missingness was independent of important observed factors such as age, gender, days post stroke, side of stroke, spasticity, and admission modified Rankin Scale [47].

One to six-cluster models were tested, with the optimum number of clusters determined using both data driven (goodness of fit indices) and pragmatic criteria (model parsimony, model interpretability). Goodness of fit indices included: Akaike’s information criterion (AIC), Bayesian information criterion (BIC), and sample size-adjusted BIC (ssBIC) with lower scores indicating a better fitting model [13]. Likelihood ratio tests (Vuong-Lo-Mendell-Rubin Likelihood Ratio Test, Lo-Mendell-Rubin Adjusted Ratio Test) determined if a model with *k* clusters was favoured over the model with *k-1* clusters [48,49]. Model pairs were tested until no further improvement in model fit occurred (*p*-value ≥0.05). Pragmatic fit criteria included acceptable entropy (>0.8) [50], high probability of belonging to each cluster (≥0.9), and potential clinical interpretability which included a minimal cluster size of 10% (7 participants) [51,52]. A post-hoc sensitivity analysis was conducted to assess integrity of the final model (e.g. changes to cluster membership, model fit) when outliers were removed from the model (*n* = 4 participants who sustained an extension of their stroke during the first four weeks of admission). We define a stroke extension as a period of improvement or stabilization for at least 24 h after the initial stroke, followed by a subsequent lesion extension and neurological worsening [53].

#### Comparison of characteristics between trajectories

Once the optimal number of clusters and membership to each cluster was established, regression analyses were conducted to determine any difference in participant characteristics across the four clusters by comparison to a reference cluster (identified as having the lowest progression based on Box and Block Test change scores). Baseline variables tested included age (in years), male gender, lesioned hemisphere, stroke sub type (ischaemic versus haemorrhagic), co-morbidity, admission Upper Limb-Motor Assessment Scale score, admission Manual Muscle Test score, admission Box and Block Test score, as well as the presence of spasticity. Other variables collected during admission included length of stay and repetitions of arm exercise completed in the first week. Weekly repetitions of arm exercise were also plotted against Box and Block test scores for individuals in each cluster using bubble plot graphs.

## Results

### Participant characteristics

Box and Block Test scores were available for 100% (*n* = 74) of participants at baseline, 100% (*n* = 74) at 1 week, 84% (*n* = 61) at 2 weeks, 58% (*n* = 43) at 3 weeks and 43% (*n* = 32) at 4 weeks. Missing Box and Block Test data were due to participants being discharged during the study period. One participant died prior to week 4. The pattern of missing data satisfied the missing at random (MAR) assumption (*n* = 74, χ^2^ distance = 66, d.f.= 63, *p* = 0.382), so were unlikely to bias model estimation [54].

Participant baseline characteristics are presented in Table 1. The average age was 75.1 years (standard deviation (SD) 12.5). Participants had a moderately severe level of disability as indicated by a median modified Rankin Scale score of 4.5 (IQR, 1.0). Participants’ stroke unit length of stay ranged from 3 to 124 days, with an average of 34.1 days (SD 27.5, median 23.0, IQR 36.5). Table 2 presents participant characteristics by cluster for the final (4-cluster) model.

**Table 1.** Characteristics of study participants (*n* = 74).

Characteristic	*N* (%)	Mean (SD)	Median (IQR)	Range	Recoded for regression analysis
Age (y)		75.1 (12.5)	78 (18.8)	39–98	
Males	42 (56.8)	–	–	–	0 If female; 1 if male
Type of stroke					
Right sided lesion	41 (55.4)	–	–	–	0 If left; 1 if right
Left sided lesion	33 (44.6)	–	–	–	
Ischaemic	63 (85.1)	–	–	–	0 If haemorrhagic; 1 if ischaemic
Haemorrhagic	11 (14.9)	–	–	–	
Days post-stroke	–	2.6 (6.1)	0.0 (1.0)	0–33	
Length of inpatient stay (days)	–	34.1 (27.5)	23.0 (36.5)	3–124	
Admission modified Rankin Scale (0–5)	–	4.4 (0.7)	4.5 (1.0)	3–5	
Severe disability (mRS >4)		37 (50.0)			
Charlson Comorbidity Index	–	1.4 (1.5)	1.0 (2.0)	0–6	0 If CCI ≤ 1; 1 if CCI >1
Six Item Screener (0–6)	–	4.8 (0.7)	5.0 (1.0)	4–6	
Box and Block Test	–	16.6 (14.1)	14.5 (28.5)	0–47	
Upper Limb Motor Assessment Scale (0–18)	–	9.6 (5.7)	12.0 (1.0)	0–17	
Action Research Arm Test (0–57)	–	30.8 (19.6)	37.5 (34.8)	0–57	
Manual Muscle Test (0–30)	–	22.3 (7.0)	24.0 (8.5)	5–30	
Repetitions completed in week 1		160 (296.9)	60.0 (152.0)	0–1635	

SD: standard deviation; M: median; IQR: interquartile range.

**Table 2.** Characteristics for each cluster in the 4-cluster model and comparisons between clusters.

Characteristic	All clusters combined	Cluster 1	Cluster 2	Cluster 3	Cluster 4
		(Reference) LOWstart/LOWprogress	MODstart/LOW progress	LOWstart/HIGHprogress	MODstart/MODprogress
	(*n* = 74)	(*n* = 19)	(*n* = 15)	(*n* = 15)	(*n* = 25)
Age (years) mean, SD	75.1 (12.5)	75.7 (14.6)	75.9 (10.7)	76.6 (12.8)	73.4 (12.1)
*β* (95% CI)	–	–	0.25 (−8.49 to 8.98)	0.91 (−7.82 to 9.65)	−2.28 (−9.98 to 5.41)
Males *N* (%)	42 (56.8)	10 (52.6)	8 (53.3)	10 (66.7)	14 (56.0)
OR (95% CI)	–	–	1.03 (0.26 to 3.99)	1.80 (0.44 to 7.31)	1.15 (0.35 to 3.79)
Type of stroke					
Right sided lesion					
*N* (%)	41 (55.4)	9 (47.4)	8 (53.3)	8 (53.3)	8 (32.0)
OR (95% CI)	–	–	1.27 (0.33 to 4.93)	1.27 (0.33 to 4.93)	0.52 (0.15 to 1.79)
Ischaemic *N* (%)	63 (85.1)	16 (84.2)	13 (86.7)	12 (80.0)	22 (88.0)
OR, 95% CI	–	–	1.22 (0.18 to 8.42)	0.75 (0.13 to 4.39)	1.38 (0.24 to 7.72)
Days post-stroke					
Mean (SD)	2.6 (6.1)	4.9 (9.4)	1.7 (4.5)	0.1 (0.4)	2.7 (5.1)
β (95% CI)	–	–	−3.21 (−7.34 to 0.91)	−4.81 (−8.94 to −0.69)*	−2.23 (−5.86 to 1.41)
Admission modified Rankin Scale (0–5)					
Mean (SD)	4.4 (0.7)	4.7 (0.6)	4.3 (0.6)	4.5 (0.6)	4.0 (0.8)
β (95% CI)	–	–	−0.40 (0.87 to 0.0)	−0.27 (0.74 to 0.20)	−0.74 (−1.15 to −0.32)*
Severe disability (mRS = 5)					
*N* (%)	37 (50.0)	15 (78.9)	6 (40.0)	8 (53.3)	8 (32.0)
OR (95% CI)	–	–	0.18 (0.39 to 0.81)*	0.30 (0.68 to 1.36)	0.13 (0.31 to 0.50)*
Charlson Comorbidity Index					
Mean (SD)	1.4 (1.4)	1.4 (1.7)	1.6 (1.4)	2.0 (1.7)	1.0 (1.4)
β (95% CI)	–	–	0.30 (−0.77 to 1.36)	0.43 (−0.63 to 1.50)	−0.21 (−1.15 to 0.73)
Six Item Screener (0-6)					
Mean (SD)	4.8 (0.7)	4.6 (0.6)	5.1 (0.8)	4.7 (0.7)	4.9 (0.8)
β (95% CI)	–	–	0.49 (−0.02 to 1.00)	0.15 (−0.36 to 0.66)	0.34 (−0.11 to 0.79)
Spasticity *N* (%)	10 (13.5)	4 (21.5)	2 (13.3)	3 (20.0)	1 (4.0)
OR (95% CI)	–	–	0.58 (0.90 to 3.70)	0.94 (0.18 to 5.02)	0.16 (0.16 to 1.53)
Box and Block Test Mean (SD)					
β (95% CI)	16.6 (14.1)	1.4 (3.8)	25.0 (4.1)	6.3 (4.9)	29.4 (10.2)
	–	–	23.58 (18.82 to 28.34)*	4.85 (0.85 to 9.61)*	28.02 (23.82 to 32.21)*
Upper Limb Motor Assessment Scale					
Mean (SD)	9.6 (5.7)	3.3 (4.1)	12.8 (3.0)	6.6 (3.9)	14.1 (2.7)
*β* (95% CI)	–	–	9.60 (7.24 to 11.97)*	3.34 (0.97 to 5.70)*	10.86 (8.77 to 12.94)*
Action Research Arm Test					
Mean (SD)	30.8 (19.6)	9.1 (14.6)	40.2 (7.8)	21.8 (13.4)	47.1 (10.7)
β (95% CI)	–	–	31.09 (22.86 to 39.33)*	12.69 (4.46 to 20.93)*	38.01 (30.76 to 45.27)*
Manual Muscle Test					
Mean (SD)	22.3 (7.0)	14.9 (8.1)	25.5 (3.6)	21.1 (5.3)	26.8 (2.3)
β (95% CI)	–	–	10.64 (7.07 to 14.21)*	6.24 (2.67 to 9.81)*	11.87 (8.72 to 15.01)*
Arm repetitions completed in week 1					
Mean (SD)	160 (296.9)	262.2 (418.3)	120.7 (165.9)	145.0 (190.2)	235.0 (403.2)
OR (95% CI)	–	–	−141.49 (−373.52 to 90.54)	−116.96 (−348.99 to 115.07)	−26.96 (−231.42 to 177.50)
Length of inpatient stay (days)					
Mean (SD)	34.1 (27.5)	56.5 (35.3)	25.3 (20.6)	34.8 (21.0)	22.0 (15.9)
OR (95% CI)	–	–	−31.19 (−47.79 to −14.59)*	−21.73 (−38.33 to −5.13)*	−34.72 (−49.35 to −20.10)*

Data presented with mean (SD) or number (%). SD standard deviation; *β* regression coefficient; OR odds ratio. Clusters 2–4 were compared with cluster 1. * indicates p-value was significant.

### Latent class growth analysis (selection of ideal number of clusters)

**Figure 1.**
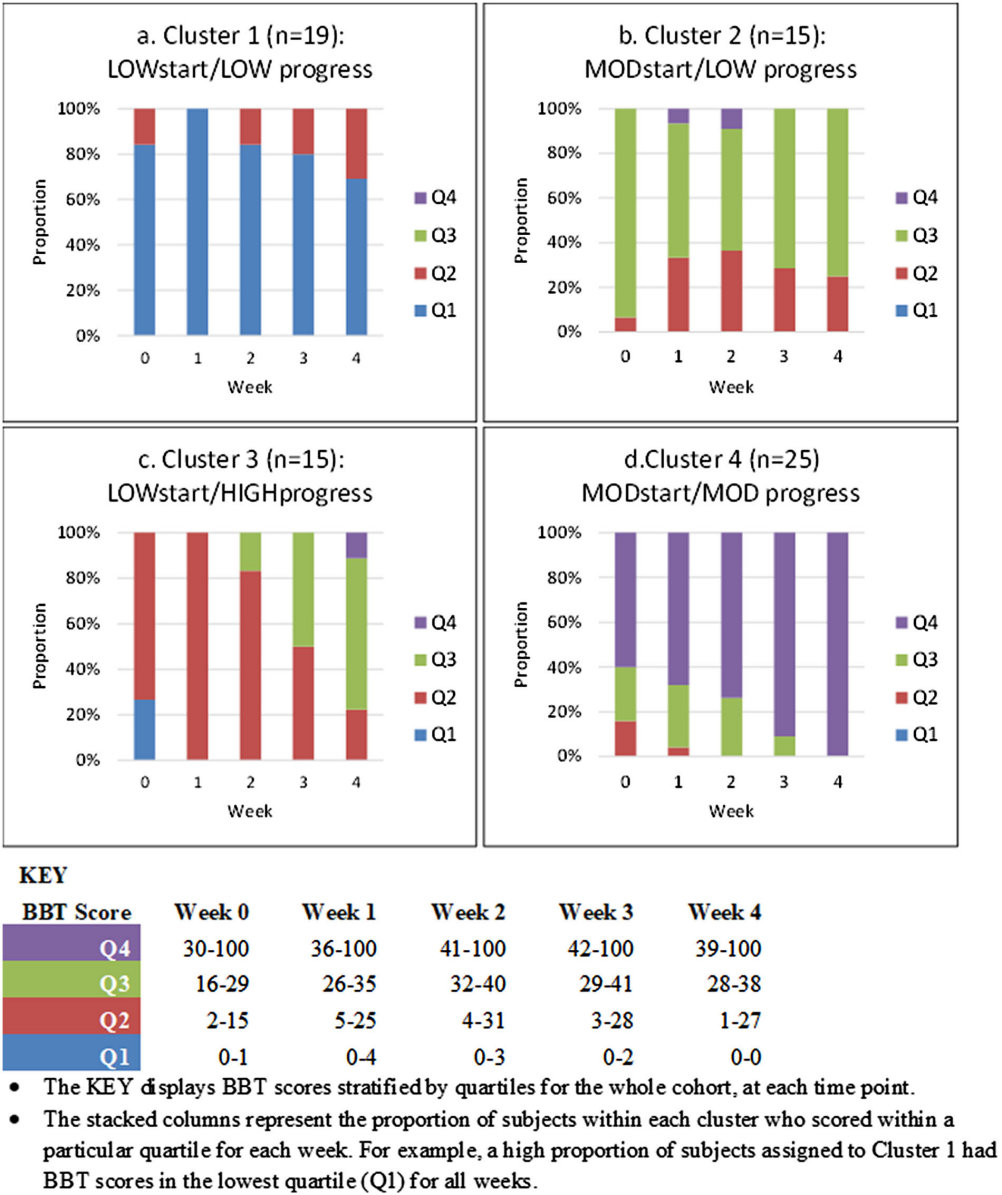
Proportion of subjects with Box and Block Test scores stratified by quartile (Q1 - Q4) at each time point for each of the 4 clusters. • The KEY displays BBT scores stratified by quartiles for the whole cohort, at each time point. • The stacked columns represent the proportion of subjects within each cluster who scored within a particular quartile for each week. For example, a high proportion of subjects assigned to Cluster 1 had BBT scores in the lowest quartile (Q1) for all weeks.

The 1-cluster to 6-cluster goodness of fit indices are presented in Table 3. Linear growth curves were fitted for all models. Higher order curves (e.g. quadratic) were not considered given that some participants were discharged prior to week 4. When all models were considered, the 4-cluster LCGA model was chosen based on the best combination of fit indices (AIC = 547.3, ssBIC = 536.3), likelihood ratio tests (VLMR LRT *p* = 0.101, LMR LRT *p* = 0.116), high average posterior probability of belonging (range 0.92–0.99), and high entropy (0.86). In addition, the 4-cluster model was the most parsimonious solution to describe (potentially) clinically distinct trajectories. Each cluster was labelled based on the start and progress scores on the Box and Block Test. Figure 1 shows the proportion of participants’ Box and Block Test score within each quartile (0–25%, 26–50%, 51–75%, 76–100%) for each time point in the 4-cluster model.

**Table 3.** Goodness of fit criteria for 2-cluster to 6-cluster models.

Fit indices	1-Cluster	2-Cluster	3-Cluster	4-Cluster	5-Cluster	6-Cluster
	Model	Model	Model	Model	Model	Model
Log likelihood	−393.1	−309.4	−269.5	−260.6	−252.7	−248.4
AIC	794.3	632.9	559.1	547.3	537.5	534.8
BIC	803.5	649.1	582.2	577.2	574.4	578.5
ssBIC	790.9	627.0	550.6	536.3	524.0	518.7
VLMR LRT (*p*-value)	–	<0.001	0.002	0.101	0.427	0.488
LMR LRT (*p*-value)	–	<0.001	0.003	0.116	0.446	0.506
Entropy	–	0.97	0.93	0.86	0.90	0.90
Posterior probabilities (range)	1.00	0.99–0.99	0.95–0.99	0.92–0.99	0.90–0.99	0.85–0.97
Cluster membership (C1/C2/C3…)	74	55/19	32/19/23	19/15/15/25	19/23/16/12/4	4/13/23/17/10/7

AIC: Akaike information criterion; BIC: Bayesian information criterion; ssBIC: Sample size adjusted Bayesian information criterion; VLMR LRT: Vuong-Lo-Mendell-Rubin likelihood ratio test; LMR LRT: Lo-Mendell-Rubin adjusted likelihood ratio test.

**Figure 2.**
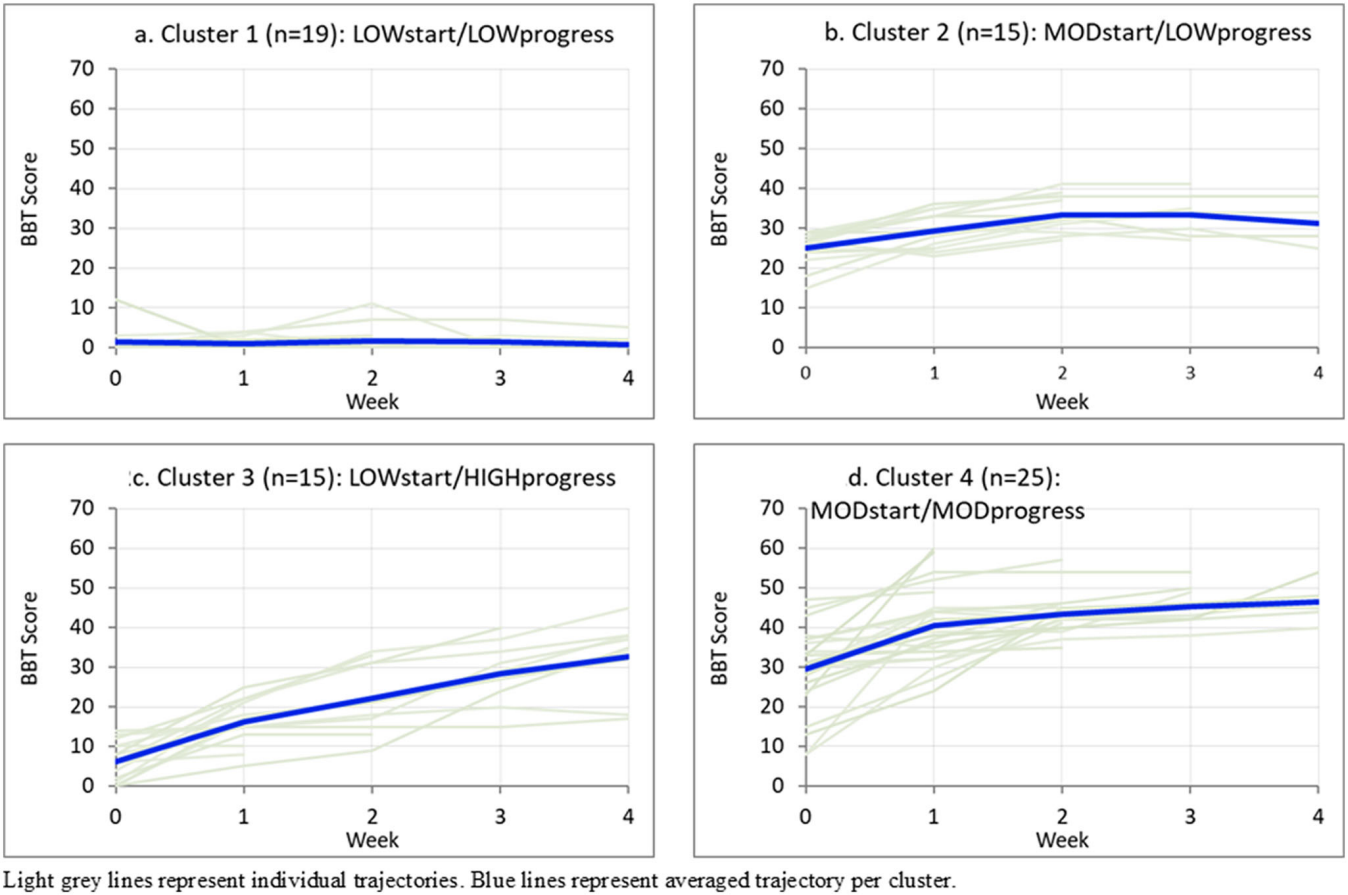
Weekly Box and Block Test scores by cluster. Light grey lines represent individual trajectories. Blue lines represent averaged trajectory per cluster.

Cluster 1 comprised 19 participants (26%) with low baseline arm function who showed little/no overall recovery over four weeks (‘LOWstart/LOWprogress’). Cluster 2 comprised 15 participants (20%) with moderate baseline arm function who also showed minimal recovery (‘MODstart/LOWprogress’). Cluster 3 comprised 15 participants (20%) with low baseline arm function who showed a high level of recovery (‘LOWstart/HIGHprogress’). Cluster 4 comprised 25 participants (34%) with moderate arm function who showed a moderate level of recovery (‘MODstart/MODprogress’) (Figure 2).

Sensitivity analysis was conducted with four outliers retained and removed from the 4-cluster model (*n* = 4 participants who had an extension of their stroke). Class assignment remained identical for remaining cases (i.e. there was no shift in cluster membership), and there was negligible change in model fit.

### Associations between participant characteristics and cluster membership

Baseline characteristics of participants in Clusters 2, 3, and 4 were compared against participants in Cluster 1: LOWstart/LOWprogress (reference cluster) (see Table 2). On average, participants in the LOWstart/HIGHprogress cluster were earlier post stroke on admission (*β*: −4.81 days, 95% CI: −8.94 to −0.69) compared to the LOWstart/LOWprogress cluster. Participants in the MODstart/MODprogress cluster had lower levels of disability as indicated by modified Rankin Scale scores on admission (*β*: −0.74, 95% CI: −1.15 to −0.32) compared to the LOWstart/LOWprogress cluster. Participants in the MODstart/LOWprogress and MODstart/MODprogress clusters were less likely to have severe disability (mRS of 5) on admission (OR: 0.18, 95% CI: 0.39 to 0.81 and OR: 0.13, 95% CI: 0.31 to 0.50) respectively) compared to the LOWstart/LOWprogress cluster.

Participants in the MODstart/LOWprogress, LOWstart/HIGHprogress and MODstart/MODprogress clusters were more likely to have a higher admission score on the Box and Block Test (*β*: 23.58, 95% CI: 18.82 to 28.34; *β*: 4.85, 95% CI: 0.85 to 9.61; *β*: 28.02, 95% CI: 23.82 to 32.21 respectively), Upper Limb-Motor Assessment Scale (*β*: 9.60, 95% CI: 7.24 to 11.97; *β*: 3.34, 95% CI: 0.97 to 5.70; *β*: 10.86, 95% CI: 8.77 to 12.94 respectively), Action Research Arm (*β*: 31.09, 95% CI: 22.86 to 39.33; *β*: 12.69, 95% CI: 4.46 to 20.93; *β*: 38.01, 95% CI: 30.76 to 45.27 respectively), and Manual Muscle Test (*β*: 10.64, 95% CI: 7.07 to 14.21; *β*: 6.24, 95% CI: 2.67 to 9.81; *β*: 11.87, 95% CI: 8.72 to 15.01 respectively), compared with the LOWstart/LOWprogress cluster. On average, participants in the MODstart/LOWprogress, LOWstart/HIGHprogress and MODstart/MODprogress clusters had a shorter hospital length of stay than the LOWstart/LOWprogress cluster (*β*: −31.19, 95% CI: −47.79 to −14.59; *β*: −21.73, 95% CI: −38.33 to −5.13 and *β*: −34.72, 95% CI: −49.35 to −20.10 respectively).

### Repetitions of arm practice and cluster membership

**Figure 3.**
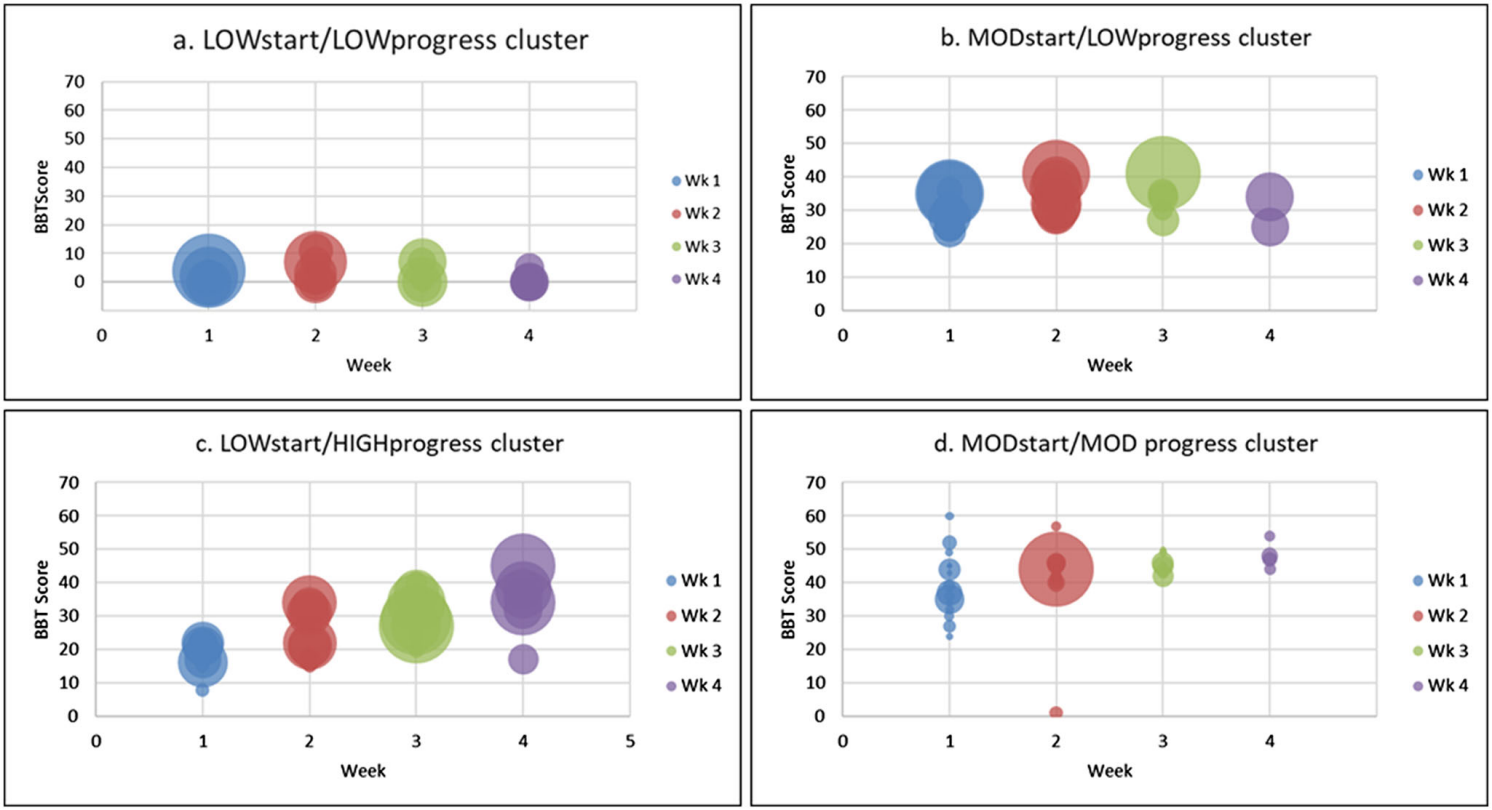
Participants Box and Block Test (BBT) scores plotted by week. Each bubble represents an individual score with bubble diameter representing number of repetitions completed per week (Overall Repetitions Range: 0–10,196; Mean (SD) 268.3 (722.1); Median (IQR): 117 (301) (greater diameter = higher number of repetitions completed).

Figure 3 shows weekly Box and Block Test scores plotted over the first month by cluster. Bubble size represents the number of repetitions of arm practice completed per participant per week (bubble diameter is proportional to number of repetitions completed). The number of repetitions completed by participants varied greatly.

## Discussion

Our study explored trajectories of early arm recovery using Box and Block Test scores collected over the first four weeks of inpatient rehabilitation. Four distinct clusters of arm recovery were identified in stroke survivors. Two clusters included participants with low to moderate levels of baseline arm function, with little to no improvement at four weeks. The remaining two clusters included participants with low to moderate levels of baseline arm function, and moderate to high rates of improvement over the study period. Compared to the LOWstart/LOWprogress cluster, participants in the LOWstart/HIGHprogress cluster were more likely to be earlier post stroke. Lower levels of disability on admission were strongly associated with the LOWstart/HIGHprogress cluster. Higher levels of baseline arm function were strongly associated with MODstart/LOWprogress, MODstart/MODprogress and LOWstart/HIGHprogress clusters when compared to the LOWstart/LOWprogress cluster.

Our study is the first to identify latent trajectories of arm recovery early after stroke using weekly outcome data. Our 4-cluster model has some similarities to PREP and PREP2 which both identified four recovery groups. In PREP2, Stinear and colleagues identified two groups that showed limited progress, known as ‘poor’ and ‘limited’, and two groups of progressors known as ‘good’ and ‘excellent’ however, they used a regression tree analysis in their study [10]. Further investigation is warranted to develop simple clinician tool using trajectories identified in our study to classify stroke survivors into likely recovery groups and predict recovery without the need for TMS equipment or staff training. In contrast, PREP2 uses a more complex algorithm including a three-level decision tree to classify stroke survivors, based on clinical factors and a neurological biomarker namely MEPs, measured using TMS [10].

The proportional recovery model suggests that in people with non-severe motor arm impairment, the magnitude of recovery in the first 3–6 months after stroke, is approximately 0.7 times the initial impairment measured with Fugl Myer assessment [9]. The model also suggests that 30% of people with severe arm impairment do not show such recovery (‘non responders’) [9]. In our study a high proportion (*n* = 40; 54%) of people had severe arm impairment at baseline. Of this group 15 (37.5%) had minimal arm recovery representing the LOWstart/LOWprogress group and 25 (65.5%) had high levels of recovery representing the LOWstart/high progress group. Our findings show a slightly higher proportion of ‘non responders’ at 4 weeks however we did not follow up people at 3–6 months to be able to compare to people in the proportional recovery validation studies. This comparison could be conducted in future studies with a longer follow up period.

Several models have included and used the Action Research Arm Test (SAFE and PREP [8,10,11] to measure arm recovery. We also used the Box and Block Test, which was more responsive to change than the ARAT in the first month post-stroke [55]. As with PREP and PREP2, we were unable to establish an association between arm practice dose and cluster membership [10,11]. A recent study conducted a retrospective analysis comparing the responsiveness of stroke participants who completed the 3-week Queen Square Upper Limb program - a high intensity arm training program (6 h daily, 5 days per week for 3 weeks, 90 total hours) to the Rehabilitation Gaming System - a low intensity arm training program (20–30 min/session, 3–5 days a week for 3–12 weeks, 3 to 30 total hours) across different stages of chronicity post-stroke (< 6 months to > 4 years) [56]. Stroke participants in that study who completed the high-intensity Queen Square program had superior arm outcomes to those who completed the low-intensity program at all stages post stroke [56]. Further studies are needed to investigate and model associations between type and dose of arm rehabilitation, and trajectories of recovery. For example, while the Queen Square study provides some evidence that providing arm training of different intensities produced different outcomes, future studies should investigate whether increasing rehabilitation dose (repetitions) changes a person’s arm recovery course, moving them into a more favourable cluster and/or improving their outcome.

Strengths of this study include the use of data from a consecutive sample of participants recruited to a prospective inception cohort study. Second, the use of a cohort of stroke survivors that were receiving usual inpatient rehabilitation rather than an experimental intervention. Third, the inclusion of all types of stroke with arm impairment. A final strength is use of a robust methodology including latent class growth analysis and goodness of fit indices. This approach allowed for all cases to be included, caters for missing data and outliers within the model [22]. This approach also enabled us to evaluate multiple models and determine the optimal number of clusters, reducing the potential for selection bias and misclassifying subjects into clusters. These strengths combined have resulted in the identification of arm recovery trajectories based on a robust methodology, clinical relevance and are based on a sample that is more likely to be representative of usual care.

In our study the largest improvements were observed in the first week. We acknowledge we were interested in very early recovery, and the follow-up period was relatively short. A study which follows participants over a longer period of time, for example over 12 months would potentially help us better understand if those in the poor/limited early recovery clusters continue to have the same recovery trajectory or have a slow/delayed recovery. A recent observational study by Borschmann and colleagues (2020) which followed stroke survivors over a 2-year period found the greatest amount of recovery occurred between 3 and 6 weeks with improvements still occurring at 18 months, and in some individuals at 24 months [57]. The researchers did not however perform any modelling to examine sub groups of recovery (likely due to their small sample size).

There are some other limitations to our study. First, while the sample was larger than that used in other studies [9,11], the sample size was still relatively small. A larger sample with over 100 participants per cluster would have allowed us to explore factors associated with cluster membership and outcome using multivariate statistics. Second, the original cohort study data excluded participants with limited cognition/language ability. Third, data were collected from participants at one comprehensive Australian stroke unit, and may not be generalisable to other settings. Fourth, while the Stroke Recovery and Rehabilitation Roundtable (SRRR) suggest that tools measuring change at the activity level are more likely to represent true recovery [57], we can’t be fully certain of the mechanisms behind the observed clinical improvement. Finally, the regression modelling was based on absolute assignment of participants to each cluster, instead of proportional assignment. However, the very high average probability of membership for each cluster (92%) is unlikely to impact regression estimates [58]. Because of these limitations, and the exploratory nature of the study, our findings should be interpreted with caution.

In conclusion, our findings show heterogeneity in patterns of arm recovery after stroke. Our exploratory model provides a way of classifying stroke survivors into four distinct clusters of arm recovery. The model was developed using clinical data that clinicians can easily collect at the bedside. Identification of four distinct clusters offers clinicians and stroke survivors a better understanding of the potential course of early arm recovery and can guide future model development. Further research is required in a larger cohort of stroke survivors and other rehabilitation settings to develop and validate a model that could be used by clinicians to inform early arm rehabilitation and prognosis in the rehabilitation setting.

## Data Availability

The data that support the findings of this study are available from the corresponding author, AVC, upon reasonable request.

## Appendix 1. Checklist-guidelines for reporting on latent trajectory studies (GRoLTS)

**Table:**

	Reported	Reason
1. Is the Metric of Time Used in the Statistical Model Reported?	Yes	
2. Is Information Presented About the Mean and Variance of Time Within a Wave?	No	Time structured study
3a. Is the missing data mechanism reported? Yes/No	Yes	
3b. Is a description provided of what variables are related to attrition/missing data?	Yes	
3c. Is a description provided of how missing data in the analyses were dealt with	Yes	
4. Is information about the distribution of the observed variables included?	Yes	
5. Is the software mentioned	Yes	
6a. Are Alternative Specifications of Within-Class Heterogeneity Considered? (EG LGCA vs LGMM) and clearly documented?	Yes	
6b. Are alternative specifications of Between-Class Differences in variance-covariance matrix structure considered and documented.	No	Variances and covariances were held equal across classes (default condition)
7. Are Alternative Shape and Functional Forms of the Trajectories Described?	N/A	Participant attrition on BBT score in weeks 2–4 prevented models greater than linear
8. If covariates are used, can analysis still be replicated	Yes	
9. Is information reported about the number of random start values and final iterations included?	Yes	
10. Are the model comparison tools used described?	Yes	
11. Are the Total Number of Fitted Models Reported? Including the One-Class Solution?	Yes	
12. Are the number of cases per class reported?	Yes	
13. If classification of cases in a trajectory is the goal, is entropy reported?	Yes	
14a. Is a plot included with the estimated trajectories for the Final Solution	Yes	
14b. Are Plots included with the estimated mean trajectories for each model?	Yes	
14c. Is a Plot Included of the Combination of Estimated Means of the Final Model and Observed Individual Trajectories Split out for Each Latent Class	Yes	
15. Are the characteristics of the Final Class Solution Numerically Described?	Yes	
16. Are the syntax files Available?	Yes	

MPLUS code. Title: LCGA BBT4. Data: File = LCGA.csv; Variable: Names = ID BBT_Adm BBT_Wk1 BBT_Wk2 BBT_Wk3 BBT_Wk4. BBT_AdmC BBT_Wk1C BBT_Wk2C BBT_Wk3C BBT_Wk4C; Usevariables = BBT_AdmC BBT_Wk1C BBT_Wk2C BBT_Wk3C BBT_Wk4C; Categorical = BBT_AdmC BBT_Wk1C BBT_Wk2C BBT_Wk3C BBT_Wk4C; IDVariable = ID; Missing = ALL(-999); Classes = c(4); Analysis: Type = Mixture; Starts = 500 50;! default = 20 4. PROCESSORS = 8; Model: %Overall%. i s | BBT_AdmC@0 BBT_Wk1C@1 BBT_Wk2C@2 BBT_Wk3C@3 BBT_Wk4C@4; Output: TECH1. CINTERVAL Tech11 Tech7 Entropy. PATTERNS; Savedata: file = 4 C PP.txt; save = cprobabilities.
